# Investigation into the optimal surgical conditions for coronary artery ligation for establishing a myocardial infarction model in mice

**DOI:** 10.3892/etm.2013.1154

**Published:** 2013-06-13

**Authors:** XIA YUE, HONGSHENG YU, XIALU LIN, KUI LIU, XIN WANG, FEI ZHOU, JINSHUN ZHAO, BAOBO ZOU

**Affiliations:** Medical School and Zhejiang Provincial Key Laboratory of Pathological and Physiological Technology, Ningbo University, Ningbo, Zhejiang 315211, P.R. China

**Keywords:** mice, coronary artery ligation, myocardial infarction, surgical conditions, biochemistry, histopathology

## Abstract

In the present study, the left anterior descending coronary arteries of mice under anesthesia were ligated, and the optimal surgical conditions for coronary artery ligation (CAL) in the establishment of a myocardial infarction (MI) mouse model were investigated. All mice that survived were sacrificed seven days subsequent to the successful surgery. Body weight, blood serum and heart tissues were obtained for further analysis or biochemical and histopathological examinations. The survival rate of the mice following the CAL procedure was 70%. The aspartate aminotransferase (AST), creatine kinase (CK) and lactate dehydrogenase (LDH) concentrations in the serum of the experimental mice were significantly increased compared with those of the control mice, which reflected the enzyme release from the infarcted myocardial cells. Histopathological examination showed different degrees of MI in the heart tissues of the experimental mice. The results indicate that an MI model in mice may be successfully established using CAL under the surgical conditions utilized in the present study. These conditions were cost effective and the results may be replicated by laboratories that are less well-equipped.

## Introduction

Animal models mimicking human diseases are important tools, and provide an understanding of the underlying mechanisms of many diseases. A number of surgical models that mimic human myocardial infarction (MI) have been described in recent years. However, there are very few detailed descriptions regarding the surgical conditions used when performing these surgical techniques in mice (1). At present, animals that are frequently being used to establish MI models include rats, rabbits, dogs, sheep and swine (2–7). Expense and a lack of inbred varieties limit the use of these larger animals for application in the MI models. However, the use of mice for MI models has been widely explored in recent years, due to their many advantages, such as small size, low cost and the availability of many inbred varieties. Furthermore, mice exhibit numerous similarities to human beings, including genetic, physiological, biochemical and developmental characteristics. Moreover, there are similarities in the structure of the food consumed by mice and humans (1,8,9). However, establishing a successful MI model in mice presents many challenges, since the size of the mice results in a small surgical field. This creates difficulties in exposing the left anterior descending artery, as well as in the ligation of the artery. It has been suggested that these disadvantages, in addition to the lack of standard operating procedures (SOP), may be the main reasons why mouse MI models have not been widely used in human MI research (1,10–14).

In this study, we have described suitable, cost-effective and detailed surgical conditions for coronary artery ligation (CAL), which may be used to establish an MI model in mice. These conditions have been based on previous studies, in combination with the experience of the authors in preparing an MI model in mice.

## Materials and methods

### 

#### Materials

**Figure 1.**
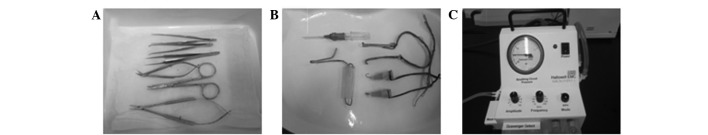
Surgical instruments and equipment. (A) Surgical instruments; (B) self-prepared surgical equipment; (C) small animal ventilator.

C57BL/6 male mice were purchased from the Shanghai Silaike Experimental Animal Company, Ltd. (license no. SCXK, Shanghai 2007-0005; Shanghai, China). An XTL continuous zoom stereomicroscope (Shenzhen Ruiwode Life Technology Company, Ltd., Shenzhen, China) and a Microvent 1 small animal ventilator (Hallowell Engineering and Manufacturing Corp., Pittsfield, MA, USA) were used in the microsurgical procedure, while a Hitachi 7600-110 autoanalyzer (Hitachi, Tokyo, Japan) was used for the biochemical analyses. Chloral hydrate and isoflurane were obtained from Sun Chemical Technology (Shanghai, China). The microsurgical instruments, endotracheal intubations and disposable intravenous catheter (22 G) used in this study are shown in Fig. 1A and B).

### Methods

#### Establishment of the MI model

A total of 30 healthy adult C57BL/6 male mice were randomly divided into two groups: control (n=10, sham group) and experimental (n=20, ligation group). Mice were housed in conditions with a controlled temperature and humidity, a 12-h light-dark cycle and free access to chow and water. The mouse experiments were approved by the Ningbo University Institutional Animal Care and Use Committee (Ningbo, China).

*Anesthesia.* Prior to the surgery, the mice were accurately weighed, and then anesthetized by intraperitoneal injection with 7% chloral hydrate (250 mg/kg body weight).

*Endotracheal intubation.* Each anesthetized mouse was placed on a flat foam board on the surgical platform and fixed with disposable tape. The mouth of the mouse was opened with a self-prepared mouth opener (Fig. 1B) and then the glottis was located with the aid of a cold light source under the stereomicroscope. An intravenous indwelling needle was inserted into the trachea along the glottis and was connected to the ventilator (Fig. 1C) with a respiratory rate of 130–140 beats/min (tidal volume of 4–5 ml). The respiratory parameters were marginally adjusted during the surgery, according to the conditions of the anesthetized animals.

**Figure 2.**
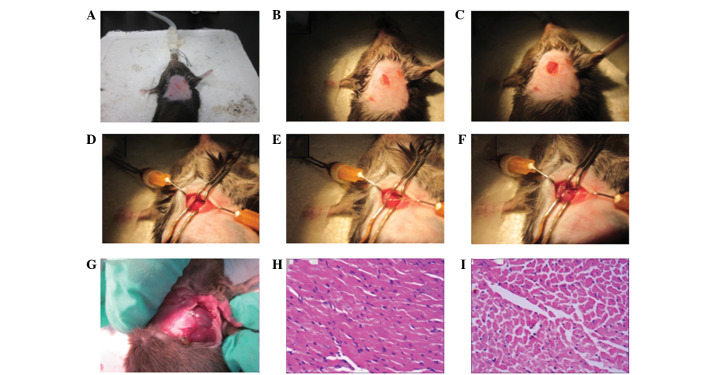
Surgical procedure and histopathological results. (A) Method for fixing the mouse; (B) skin incision; (C) chest muscle separation; (D) locating the anterior descending branch of the coronary artery; (E) capturing the anterior descending branch of the coronary artery; (F) ligating the anterior descending branch of the coronary artery. (G) Myocardial infarction (MI) under the naked-eye; (H) MI under the microscope; (I) the myocardium of control group. (H and I) Hematoxylin and eosin staining, (magnification, ×400).

*Surgical position and procedure.* The mouse was placed on the surgical platform in a right lateral position. A warm soapy water solution was used to wet the left breast fur, prior to the fur in the surgical area being scraped with a scalpel. The skin was then disinfected with 75% alcohol (Fig. 2A). Following cutting an incision (∼1 cm long) in the skin in an obliquely upward direction at the midpoint on the connection line between the lower edge of the sternum and the left armpit, a subcutaneous vein in the longitudinal direction was exposed (Fig. 2B). Blunt separation of the muscles, layer by layer, was performed with small curved forceps from the medial side of the vein along the venous direction (Fig. 2C). A self-prepared small retractor was used to fix the muscle along the left-right direction. Curved surgical scissors were used to cut an incision in the muscle (<1 cm) at 4–5 intercostals. Following this, self-prepared retractors were used to pull and open the incision up or down in a vertical direction, respectively. The retractors were fixed on the foam on the surgical platform, which left the heart fully exposed. The pericardium was then carefully lifted, separated and fixed with a retractor, prior to the retractor also being fixed (Fig. 2D).

*Coronary artery ligation: Locating the coronary artery.* The heart was initially exposed by pushing the right side of the chest gently with small curved forceps. A pink coronary artery (the left anterior descending coronary artery) was located along the edge of the left atrial appendage (Fig. 2D). In cases when the myocardial surgery took longer than expected, the coronary artery became difficult to locate. Lifting the myocardium by the suture helped expose the branch of the left coronary artery, located between the pulmonary artery cone and the left atrial appendage.

*Coronary artery ligation.* Holding a needle (7/0 G) in the right hand and using the left hand, the beating heart was stabilized with tweezers by pushing against the right ventricle. The coronary artery and a small area of the surface layer of the myocardium were then ligated with a 7/0 no-damage silk suture (Fig. 2E). To prevent the knots from coming untied, the coronary artery was wrapped twice with the silk suture before the first knot was tied. If the color of the local area surrounding the ligation and the myocardium downstream of the ligation simultaneously became gray, in addition to the movement of the corresponding myocardial region becoming minimized (Fig. 2F), the ligation was a success. These were the early signs for validating the success of the CAL procedure.

In the experimental mice, the left anterior descending coronary artery was ligated with a fast knot. In the control mice, all surgical procedures for the ligation were the same as for the experimental mice, except a slipknot was used instead of the fast knot.

*Thoracic cavity closure.* Following ligation, the self-prepared retractors were released. The heart was covered with the pericardium, and the ribs were sutured with number 5 nylon sutures. The intercostals were fully closed, in order to discharge the air from the chest. During suturing, care was taken not to damage the myocardium or the left lung. Following this, two layers of muscle were pulled together with the skin and sutured with number 5 nylon sutures. Subsequent to the removal of the breathing machine, tweezers were used to stimulate the foot of the mouse to promote spontaneous breathing. Five to ten minutes later, the endotracheal intubation was extracted, following the return of spontaneous breathing.

#### Postoperative care

Following the completion of the surgery, the mice were placed on a warm platform (32–35°C). When consciousness was completely regained, the mice were transferred into a cage to resume normal activity.

#### Biochemical and histopathological analyses

On the seventh day subsequent to surgery, the mice were weighed and then anesthetized with inhaled 2% isoflurane. Following anesthesia, blood samples were collected via the femoral artery. Serum was obtained by centrifugation at 1,150 × g for 10 min and stored at −80°C until use. The heart tissues were obtained as rapidly as possible and were rinsed in saline. Following weighing, the heart tissues were fixed in a 10% formalin solution for 24 h, embedded in paraffin and sliced. The tissue sections were stained with hematoxylin and eosin (HE) staining and observed under the microscope. The activities of aspartate aminotransferase (AST), creatine kinase (CK) and lactate dehydrogenase (LDH) in the serum were detected using the Hitachi 7600-110 autoanalyzer.

#### Statistical methods

Data are expressed as the mean ± standard deviation (SD). SPSS statistical software version 13.0 (SPSS, Inc., Chicago, IL, USA) and the Student’s t-test were used for the data analysis. P<0.05 was considered to indicate a statistically significant difference.

## Results

### 

#### Animal survival rate following surgery

The CAL procedure was performed on 30 mice, including 10 in the control group and 20 in the experimental group. Of these, five mice in the control and experimental groups, respectively, were administered 350 mg/kg body weight of 7% chloral hydrate for anesthesia. Three deaths occurred in each group due to anesthetic overdose. To all other animals, 250 mg/kg body weight of 7% chloral hydrate was administered. The five mice in the control group that were anesthetized with 250 mg/kg body weight of 7% chloral hydrate survived the procedure, while among the 15 mice anesthetized with 250 mg/kg body weight of 7% chloral hydrate in the experimental group, three deaths occurred 2–3 days subsequent to the procedure. Autopsies revealed one case with pleural effusion and two cases with cardiac rupture. The total 7-day survival rate following the surgery was 70% (seven and 14 cases in the control and experimental groups, respectively).

#### Change in animal body weight following surgery

The average preoperative weight of the mice was 29.00±3.23 g and seven days subsequent to the surgery it was 24.78±2.39 g (t=4.45, P<0.0001). This showed that the surgical trauma decreased the weight of mice significantly. Further analysis revealed no significant difference in the body weight change between the experimental and control groups.

#### MI occurrence

Evidence of MI occurrence was observed under the naked-eye (Fig. 2G) and under the microscope (Fig. 2H) in all the experimental mice. No MI was observed in the control group (Fig. 2I).

The heart weight/body weight coefficients of the control and experimental groups were (5.15±0.80)×10^−3^ and (6.52±1.13)×10^−3^, respectively, which indicated that the CAL procedure resulted in a significant enlargement of the heart (t=−2.25, P=0.0385).

#### Activities of AST, CK and LDH in the serum

The activities of AST, CK and LDH in the serum are shown in Table I. The activity levels in the serum of the ligated mice were higher than those in the control mice, which may have been a result of the release of AST, CK and LDH enzymes into the blood stream following myocardial necrosis.

## Discussion

Previous studies have demonstrated that simultaneous increases in AST, CK and LDH are frequently observed following MI (15,16). The present results showed that the serum AST, CK and LDH concentrations in the experimental mice were significantly increased compared with those in the control mice. These results indicated the onset of myocardial necrosis in the ligated mice. Using histopathological analysis, it was observed that different degrees of MI occurred in the heart tissues of the experimental mice, and the heart weight/body weight coefficient of the experimental mice was significantly higher than that of the control mice. This demonstrated that the MI model in mice was successfully established by CAL under the selected surgical conditions.

The methods for preparing an animal MI model include cryoinfarction (17–19), low amperage electrical injury (20) and CAL (9,21). Although the electrical injury and cryo-methods are simple to perform and result in relatively light damage and a constant infarct area, their clinical relevance is poor. In comparison with the electrical injury and the cryo-methods, the CAL method is more relevant to the pathological process of MI in the clinic. However, certain characteristics of mice, including their small size and low ability to resist trauma, as well as the difficulties encountered in locating the anterior descending artery and the frequent surgical complications, such as pneumothorax (22), limit the use of mice for the MI model. In this study, based on previous studies (11) and the experience of the authors (9), the aim was to optimize the surgical conditions for CAL, in order to establish an MI model in mice. The following describes the procedures used during the CAL surgery and discusses the rationale behind the selection of these procedures in the establishment of a simple, fast and successful MI model in mice.

There were several factors to consider when selecting the anesthetic agents and doses. A variety of anesthetic agents have been used in the production of an MI model in mice, including sodium pentobarbital (23), ketamine (24), chloral hydrate (25) and urethane (26). In China, sodium pentobarbital and ketamine are very expensive and complicated procedures are required in order to obtain approval prior to purchase. Chloral hydrate and urethane may be easily purchased and are cheaper than sodium pentobarbital and ketamine; as a result, they are widely used for experiments in China. In preliminary experiments, chloral hydrate and urethane were used to assess their suitability as anesthetics for the procedure. At 1,000–1,250 mg/kg body weight, urethane led to a very long duration of anesthesia (more than two hours). Following this, chloral hydrate was used to anesthetize the mice instead, which led to a fast and smooth anesthesia; however, the use of chloral hydrate also caused oral secretions in the animals. Following further studies, it was revealed that it was possible to gradually alleviate the oral secretions by reducing the anesthetic dosage. At 250 mg/kg body weight, chloral hydrate showed the optimum anesthetic results, with an anesthesia duration of 1.5–2.0 h. This fully met the surgical requirements.

In our previous experiments, sodium pentobarbital was used for the anesthesia of the mice (9). Sodium pentobarbital was more suitable for anesthesia when considering the respiratory secretion problems and spontaneous breathing recovery time. However, it was demonstrated that with the proper dosage of chloral hydrate, it was possible to eliminate the respiratory secretions. Furthermore, it was possible to minimize the recovery time for spontaneous breathing with the correct chest closure tightness. For these reasons, as well as the cost, chloral hydrate was selected over sodium pentobarbital for use as the anesthetic.

Of note is the fact that it was necessary to accurately weigh the mice prior to anesthesia and to anesthetize the mice with accurate doses. Moreover, it was desirable to administer the anesthetic drug as a single injection, since it was demonstrated that a second injection often resulted in unsatisfactory anesthetic effects. In addition, the site of intraperitoneal injection was very important. Our experience demonstrated that it was desirable to perform the anesthetic injection of chloral hydrate with a 1 ml syringe needle (26 G), piercing ∼0.5 cm into the abdominal cavity (no visible blood on the withdrawal of the needle) on the side at the midpoint of the line between the external genitalia and xiphoid. This procedure anesthetized the mice with an anesthesia induction time of 1–5 min and provided the best results for the surgery.

With regard to the securing of the mice and the intubation methods, the mice were placed in a right lateral position on the surgical platform and the lower limbs of the mice were tightly fixed. The upper limbs were loosely fixed, which alleviated muscle tension as much as possible, in order to reduce any influence on breathing.

Ventilator-assisted breathing by intubation through the mouth is a common method used during open-heart surgery when preparing an MI model in animals, since this may aid in avoiding trauma and complications, such as postoperative airway constriction caused by tracheotomy surgery, and therefore improves the quality of life of the animals. It is worth noting that intubation results in unavoidable stimulation of the larynx and airway and may damage the trachea. Occasionally, intubations may stray into the esophagus. Therefore, full exposure of the mouse glottis and a prompt, precise and delicate technique by the surgeon are all important factors for reducing respiratory complications caused by intubations, and for enhancing the survival rate of the MI model. This study demonstrated that a 22-G venous indwelling needle was suitable for tracheal intubations in mice.

With regard to the anatomical site of the surgery, the mouse was placed on the surgical platform in a right lateral position and an incision was performed along the ribs. Following the principle of blunt dissection, the dissection of the muscle was performed layer by layer. When opening the intercostal muscle to enter the chest, special attention was taken to prevent damage to the thoracic blood vessels and the heart and lungs, which may have resulted in the death of the mice. The method used in the present study did not shear the pectoralis major muscle or the ribs, which ensured that the normal anatomical structures of the chest were retained. This was beneficial for the survival of the mouse subsequent to the surgery.

Mice are very small in size and have a limited pleural area, which inevitably results in a narrow surgical field and an insufficient exposure of the surgical site. At present, there is no commercial equipment available in the Chinese domestic market for the rib distraction of mice. To resolve this, a self-prepared rib-retractor, made with a paperclip, was used, which was demonstrated to aid in exposing the surgical field during the experiments.

The surface of the mouse heart is covered by the pericardium and the thymus. The thymus, as a lymphoid organ, is particularly important for the body’s immune function. To preserve the integrity of the immune system of the mouse, the pericardium was separated and the thymus was subsequently pulled to the left side and carefully attached to a surgical retractor. Following the CAL, the thymus was placed back in its original position. In this manner, the thymus was preserved effectively. In addition, this method fully protected the left lung during the surgery.

There were numerous factors to be considered with regard to the recognition of the coronary artery and the ligation method. The blood circulation of the left ventricular myocardium is predominantly supplied by the collateral of the left anterior descending coronary. Therefore, left anterior descending coronary ligation may result in a large area of infarction in the left ventricular myocardium. The anterior descending coronary artery in the shallow myocardium is small and may be difficult to distinguish from the heart surface veins. These factors may all result in ligation failure. Therefore, the identification of the anterior descending coronary artery is a key point for a successful MI operation. The left atrial appendage is an important anatomical marker for locating the anterior descending coronary artery. With regard to the difference in the distribution direction, there are two typical distributions of the anterior descending coronary artery: The first distribution starts from the center of the left atrial appendage, while the second starts from between the center of the left atrial appendage and the pulmonary cone. It was observed that in the C57BL/6 male mice, the first distribution was more common than the second, and, therefore, the anterior descending coronary artery was most often accurately found by starting from the center and following the edge of the left atrial appendage, in the direction of the pulmonary artery cone. However, exceptions may occur in certain mice due to variations in the distribution directions of the coronary artery.

The light source of the stereomicroscope was important in the location of the anterior descending coronary artery. Under ordinary light, particularly under white light, it is difficult to distinguish between myocardium, arteries and veins. However, under a high-brightness yellow cold light source, the myocardium, arteries and veins appear bright red, pink and dark red, respectively. These three different colors were easily distinguished by the surgeons. The anterior descending coronary artery travels under the shallow myocardium. Prior to ligation, only one-third of it may be visible, with the remaining two-thirds lying beneath the myocardium. This remaining section was only observed when the anterior descending coronary artery was slightly lifted with a suture needle.

Attention is required to limit the puncture depth of the needle. The present study demonstrated that one-third to one-half of the ventricular wall penetration was a suitable depth. Ligating an appropriately sized area of myocardium and tying it securely was important, in order to prevent the tearing of the myocardium. Following the ligation of the anterior descending coronary artery, the color around the ligation point of the myocardium rapidly turned white, indicating successful ligation. However, only pathological detection was able to serve as the final and gold standard for a successful MI ligation.

An additional factor to be considered was the prevention, identification and treatment of pneumothorax. Pneumothorax is a common complication in the modeling process and may frequently result in the death of the mice following the surgical wound closure. The tolerance of mice to pneumothorax is poor, due to their small pleura. It was demonstrated that incomplete air exhaustion prior to chest closure was the primary reason for the pneumothorax. By increasing the tidal volume to expand the lung lobe and performing an appropriate repeated chest-squeezing to drain the residual air in the thoracic cavity, the incidence of the pneumothorax may be significantly reduced. A failure to tightly close the chest cavity following surgery may lead to air exhaustion failure. Therefore, during muscle separation in the thoracotomy, it was necessary to focus on reducing muscle injury, in order to enhance postoperative chest tightness. Following chest closure, the majority of the mice were able to restore spontaneous breathing without prolonged mechanical ventilation. However, there were a few mice that developed the symptoms of pneumothorax. Therefore, it was considered desirable for the endotracheal intubations to remain for 5–10 min following chest closure, in order to prevent the occurrence of pneumothorax.

With regard to the administration of antibiotics following surgery, certain studies of MI models have (11) suggested that an intramuscular injection of penicillin be used to prevent postoperative infection. It was demonstrated in the present study that surgical instrument sterilization and disinfection with 75% alcohol during the procedure was a suitable method of preventing infection.

Maintaining the warmth of the animals during and following the surgery was crucial, as a complete procedure to establish an MI model normally lasted 1–2 h. This was due to the fact that while the surgery itself may only take 20–30 min, the mice required 1–2 h to wake following the surgery, due to the anesthesia.

Following the surgery, the recovering anesthetized mice were placed in a cage. The cage was then placed on a warm thermostatic plate (30–35°C) until the animals regained consciousness.

In conclusion, the results of the study showed that the mouse MI model prepared by ligation of the anterior descending coronary artery under the selected surgical conditions was reliable, cost effective and successful. The procedure has been described in detail to enable easy and effective replication, even in less well-equipped labs. Further studies are required to simplify, optimize and standardize the surgical conditions for preparing a mouse MI model to facilitate the wider use of the mouse MI model in MI research.

## Figures and Tables

**Table I.** Activities of AST, CK and LDH in the serum.

Detection indicators	Group	No. of animals	Value (U/l, 10^−3^)	P-value
AST	Control	7	56.70±14.19	0.048^a^
	Experimental	14	89.32±40.04	
LDH	Control	7	293.33±64.66	0.024^a^
	Experimental	14	470.73±185.92	
CK	Control	7	45.33±23.09	0.027^a^
	Experimental	14	98.23±41.44	

Values are presented as mean ±standard deviation.

a Significantly different compared with control. AST, aspartate aminotransferase; CK, creatine kinase; LDH, lactate dehydrogenase.
